# Nomogram to predict rapid kidney function decline in population at risk of cardiovascular disease

**DOI:** 10.1186/s12882-022-02696-9

**Published:** 2022-02-10

**Authors:** Qiuxia Zhang, Junyan Lu, Li Lei, Guodong Li, Hongbin Liang, Jingyi Zhang, Yun Li, Xiangqi Lu, Xinlu Zhang, Yaode Chen, Jiazhi Pan, Yejia Chen, Xinxin Lin, Xiaobo Li, Shiyu Zhou, Shengli An, Jiancheng Xiu

**Affiliations:** 1 Department of Cardiology, Nanfang Hospital, Southern Medical University, No. 1838, Guangzhou Avenue North, Guangzhou, 510515 China; 2 Department of Cardiology, Zhengcheng Branch of Nanfang Hospital, Zengcheng District, Guangzhou, China; 3 Community Health Service Center, Zengjiang Avenue, Zengcheng District, Guangzhou, China; 4 Department of Public health, Xintang Hospital, Zengcheng District, Guangzhou, China; 5 Department of Biostatistics, School of Public Health, Southern Medical University, No. 1838, Guangzhou Avenue North, Guangzhou, 510515 China

**Keywords:** Risk prediction model, Rapid kidney function decline, Cardiovascular disease

## Abstract

**Background:**

To develop a reliable model to predict rapid kidney function decline (RKFD) among population at risk of cardiovascular disease.

**Methods:**

In this retrospective study, key monitoring residents including the elderly, and patients with hypertension or diabetes of China National Basic Public Health Service who underwent community annual physical examinations from January 2015 to December 2020 were included. Healthy records were extracted from regional chronic disease management platform. RKFD was defined as the reduction of estimated glomerular filtration rate (eGFR) ≥ 40% during follow-up period. The entire cohort were randomly assigned to a development cohort and a validation cohort in a 2:1 ratio. Cox regression analysis was used to identify the independent predictors. A nomogram was established based on the development cohort. The concordance index (C-index) and calibration plots were calculated. Decision curve analysis was applied to evaluate the clinical utility.

**Results:**

A total of 8455 subjects were included. During the median follow-up period of 3.72 years, the incidence of RKFD was 11.96% (*n* = 1011), 11.98% (*n* = 676) and 11.92% (*n* = 335) in the entire cohort, development cohort and validation cohort, respectively. Age, eGFR, hemoglobin, systolic blood pressure, and diabetes were identified as predictors for RKFD. Good discriminating performance was observed in both the development (C-index, 0.73) and the validation (C-index, 0.71) cohorts, and the AUCs for predicting 5-years RKFD was 0.763 and 0.740 in the development and the validation cohort, respectively. Decision curve analysis further confirmed the clinical utility of the nomogram.

**Conclusions:**

Our nomogram based on five readily accessible variables (age, eGFR, hemoglobin, systolic blood pressure, and diabetes) is a useful tool to identify high risk patients for RKFD among population at risk of cardiovascular disease in primary care. Whereas, further external validations are needed before clinical generalization.

**Supplementary Information:**

The online version contains supplementary material available at 10.1186/s12882-022-02696-9.

## Introduction

Chronic kidney disease (CKD) is an increasingly serious public health problem [1], with the number of patients with reduced estimated glomerular filtration rate (eGFR) increased by 70% globally from 1990 to 2016 [2]. Rapid kidney function decline (RKFD) is associated with cardiovascular disease (CVD) [3], incident CKD [4] and all-cause mortality [5]. However, due to their comorbidities, individuals at risk of cardiovascular disease usually need to take antihypertension medicine, antidiabetic drugs or antithrombotic agents, which may increase the burden on the kidney. Therefore, early identifying and treating the individuals with high risk of RKFD may help decrease the incidence of cardiovascular events among population at risk of CVD [6].

Previous studies had established several effective prediction models to identified patients at risk of worsening renal function. However, the endpoint they mainly focused on was incident CKD which was defined as incident eGFR less than 60 ml/min/1.73m^2^ [7, 8]. Risk prediction models constructed to identify patients at risk of incident CKD were only applicable for individuals without baseline CKD. Once the patient had progressed to CKD, these prediction models were no longer applicable. In this situation, risk prediction model to identify patients at risk of RKFD, which was defined as a relative fall in eGFR, may be more practical. Therefore, in the present study, we aimed to develop and validate a prediction model to quickly identify individuals at risk of RKFD among population at risk of cardiovascular disease.

## Methods

### Study design and participants

**Fig. 1:**
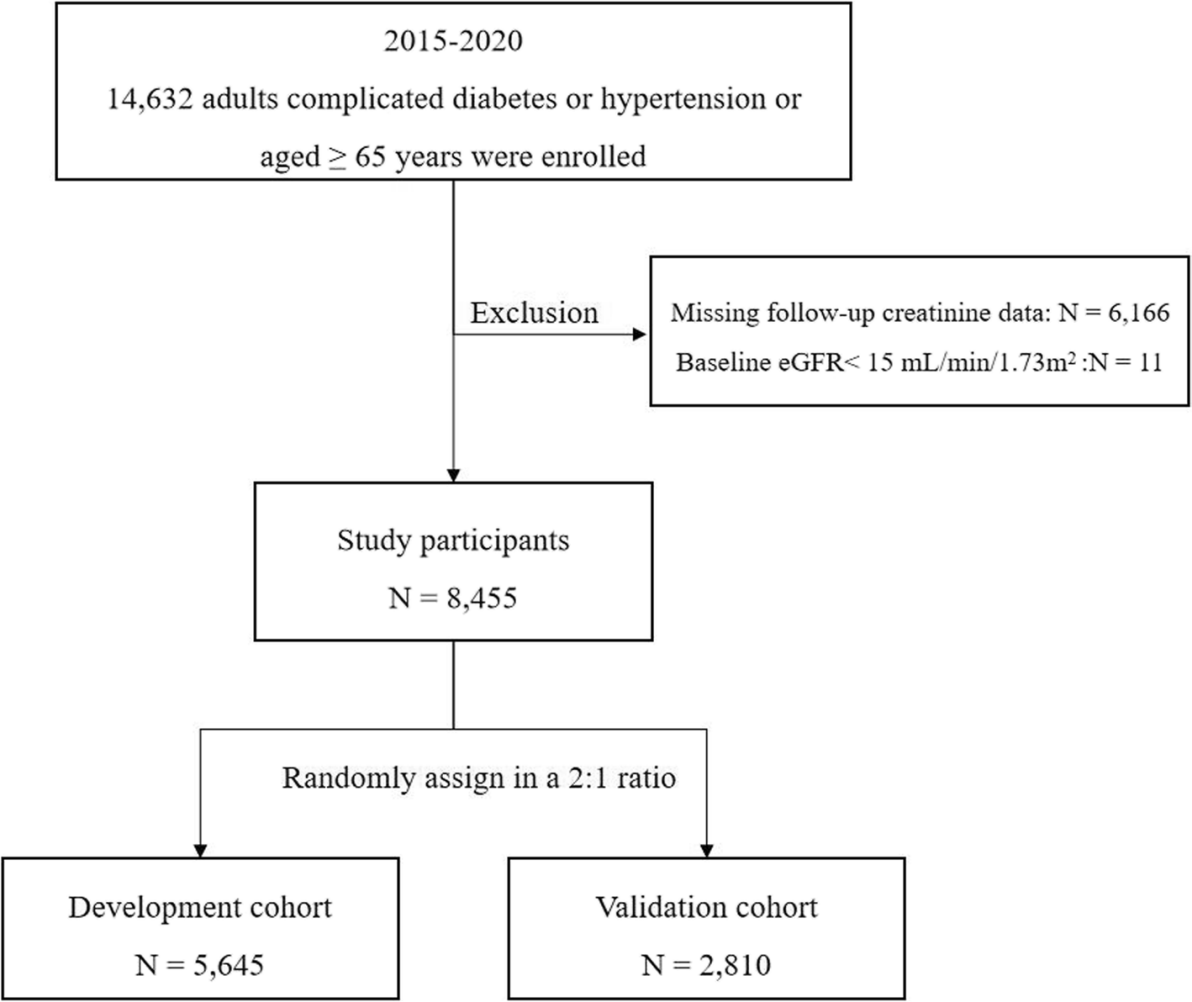
Flow chart of study design and participants. eGFR: estimated glomerular filtration rate

Residents who participated in the annual community physical examination of China National Basic Public Health Service in Guangzhou, Guangdong between January 2015 and December 2020 were included. According to the policy, to participate in the Basic Public Health Service, one must be at risk of cardiovascular disease, which was defined as having at least one of the following risk factors: 1. elder (age ≥ 65 years); 2. having hypertension; 3. having diabetes mellitus. In the current analysis, we excluded the following participants: 1) pre-existing end-stage kidney disease (eGFR < 15 mL/min/1.73m^2^), and 2) missing follow-up creatinine data. The study was performed in accordance with the Declaration of Helsinki and was approved by the Ethics Committee of the Nanfang Hospital (NFEC-2021-083). All the included participants were randomly assigned to a development cohort and a validation cohort in a 2:1 ratio (Fig. 1).

### Procedures

The Modification of Diet in Renal Disease (MDRD) equation was used to calculate eGFR [9]. Serum creatinine was measured by enzyme method. Hypertension was defined as systolic blood pressure (SBP) ≥140 mmHg, diastolic blood pressure (DBP) ≥90 mmHg, or use of antihypertensive medications. Diabetes mellitus was defined as random blood glucose level ≥ 11.1 mmol/L or fasting plasma glucose level ≥ 7.0 mmol/L or hemoglobin A1c (HbA1c) ≥6.5%. Body mass index (BMI) values calculated by the body weight and height of each participant as the follow equation: BMI = weight (kg)/[height (m)]^2^. Smoking was classified as ever smoking vs never smoking. Exercise habit was acquired through questionnaires (http://www.nbphsp.org.cn/jbgw/lnr/), which was designed by the Health Department of National Health Commission and included the following two questions: “How often do you exercise?”; “What kinds of exercise do you usually have?”

### Endpoint and follow-up

The endpoint of this study was the performance of RKFD prediction model. RKFD was defined as the reduction of eGFR ≥40% (3,17) during follow-up period. Participants without RKFD during the whole follow-up period were defined as event-free. After enrollment, laboratory measures and medical history were annually performed and collected during the follow-up period.

### Patient and public involvement

The study was a retrospective cohort study. Health records were obtained from regional chronic disease management platform of Zengcheng District of Guangzhou for all included patients. And we appreciate all participants for their valuable contribution.

### Statistical analysis

The unpaired, 2-tailed t test was used to analyse quantitative variables that were normally distributed and homoscedastic. These variables were summarized as mean ± SD. The Wilcoxon rank-sum test was used to analyse quantitative variables that were non-normally distributed or not homoscedastic, and these variables were summarized as median (interquartile range). Qualitative variables such as gender, comorbidities, life style, and medications were compared using the χ^2^ test or Fisher’s exact test and were summarized as percentages.

The multivariable Cox regression analysis was used to determine the risk factors of rapid eGFR decline. Variables with less than 15% missing values and were imbalanced between groups in the development cohort or that are clinically important were included in the univariable Cox regression analysis. Variables with significance in the univariate analysis were preliminarily screened out and to be included in the multivariable Cox regression analysis. For the determination of significant variables, *P* < 0.05 was the threshold. And we manually investigated the contribution of the remaining variables to determine the final predictors. Then, the risk prediction nomogram was formulated based on the results and by using the rms package of R. To form the nomogram, each regression coefficient in the multivariable Cox regression was proportionally converted into a 0- to 100-point scale. The variable with the highest β coefficient (absolute value) was assigned 100 points. The points are added across each variable to calculate the total points, which are finally converted to predicted probabilities.

The nomogram was evaluated in both the development and validation cohorts. Discriminative ability was assessed using the concordance index (C-index) and the area under the time-dependent receiver operating characteristic curve (AUC). Calibration was assessed using a bootstrap approach with 1000 resamples to compare the predicted event rate with the observed one in the study. And the decision curve analysis was applied to evaluate the clinical utility of the nomogram. The entire cohort was also divided into low-risk group (≤ 150 points) and high-risk group (> 150 points), and the event rate was also compared between groups. Missing data were not imputed. In all analyses, *P* < 0.05 was considered statistically significant. All analyses were conducted with R software (version 4.0.3; R Foundation for Statistical Computing, Vienna, Austria) and SPSS (version 26.0).

## Results

### Population characteristics

The study design is shown in the Fig. 1. After excluding those with baseline eGFR <15 mL/min/1.73m^2^ (*n* = 11) or missing follow-up creatinine data (*n* = 6166), 8455 participants at risk of cardiovascular disease were finally included in the study. Five thousand six hundred forty-five and 2810 participants were further categorized into the development and validation cohorts, respectively (Table S1). During the median follow-up period of 3.72 years, the incidence of RKFD was 11.96% (*n* = 1011), 11.98% (*n* = 676) and 11.92% (*n* = 335) in the entire cohort, the development cohort and the validation cohort, respectively. Participants developed RKFD approximately two-third were female (68.8%), and the mean age was 67.66 ± 6.70 years. No significant differences were identified between the development and validation cohorts.

Table 1 shows the basic characteristics of the study participants in the development cohort. Compared with those without RKFD, the participants who developed RKFD were more likely to be female and to have a history of type 2 DM and hypertension, to be taking metformin for DM. They were also older and had a higher SBP but not a higher DBP. As for laboratory examinations, lower hemoglobin, serum creatinine, cholesterol, and uric acid (UA), and higher eGFR were found among those developing RKFD, whereas they were less likely to be ever smokers or drinkers.

**Table 1:** Baseline characteristics of participants with and without follow-up RKFD in the development cohort

Variables	Missing data (%)	RKFD (n = 676)	Non-RKFD (***n*** = 4969)	***P*** value
Age,y	0 (0.00)	68.31 ± 6.84	67.57 ± 6.67	< 0.010
Female, n (%)	0 (0.00)	465 (68.8)	3003 (60.4)	< 0.001
Height (cm)	0 (0.05)	154.31 ± 7.44	156.10 ± 8.08	< 0.001
Weight (kg)	4 (0.07)	58.80 ± 10.49	59.61 ± 10.31	0.058
BMI (kg/m^2^)	4 (0.07)	24.64 ± 3.73	24.42 ± 3.57	0.128
Waist (cm)	27(0.48)	84.96 ± 9.03	84.94 ± 9.46	0.954
SBP (mmHg)	11 (0.19)	150.41 ± 20.72	145.62 ± 20.29	< 0.001
DBP (mmHg)	15 (0.27)	81.21 ± 11.58	81.79 ± 11.29	0.217
Diabetes mellitus, n (%)	0 (0.00)	196 (29.1)	894 (18.6)	< 0.001
Hypertension, n (%)	0 (0.00)	557 (82.4)	3675 (74.0)	< 0.001
Ever smoking, n (%)	1 (0.02)	79 (11.7)	926 (18.6)	< 0.001
Ever drinking, n (%)	0 (0.00)	48 (7.1)	630 (12.7)	< 0.001
Exercise	4 (0.07)			< 0.001
Never, n (%)		356 (52.7)	2239 (45.1)	
Once a week, n (%)		152 (22.5)	727 (14.6)	
Few times a week, n (%)		28 (4.1)	261 (5.3)	
Daily, n (%)		140 (20.7)	1738 (35.0)	
Laboratory examination				
Fasting glucose (mmol/L)	6 (0.11)	4.72 [4.26, 5.52]	4.88 [4.43, 5.50]	0.003
RBC (10^12^/L)	90 (0.16)	4.63 [4.28, 5.08]	4.64 [4.33, 5.01]	0.790
Hemoglobin (g/L)	57(1.01)	134.00 [124.00, 143.00]	137.00 [127.00, 146.00]	< 0.001
WBC (10^9^/L)	27 (0.48)	6.60 [5.70, 7.60]	6.59 [5.60, 7.70]	0.357
PLT (10^9^/L)	150 (2.66)	209.00 [174.25, 247.00]	211.00 [177.00, 250.00]	0.259
ALT (U/L)	10(0.18)	23.60 [17.80, 32.65]	22.50 [17.20, 30.20]	0.001
BUN (mmol/L)	23 (0.41)	5.10 [4.40, 6.20]	5.50 [4.60, 6.40]	< 0.001
Cholesterol (mmol/L)	13 (0.41)	5.20 [4.46, 5.91]	5.35 [4.64, 6.11]	< 0.001
Triglyceride (mmol/L)	11 (0.19)	1.38 [0.96, 2.02]	1.42 [0.99, 2.08]	0.262
Uric acid (umol/L)	1146(20.3)	323.90 [261.30, 404.00]	375.15 [304.72, 452.10]	< 0.001
Scr (umol/L)	0 (0.00)	54.45 [45.80, 68.10]	67.70 [56.40, 81.80]	< 0.001
eGFR (mL/min/1.73m^2^)	0 (0.00)	112.82 [91.70, 136.01]	90.60 [76.12, 106.36]	< 0.001
Medications				
ACEI/ARB, n (%)	321 (5.69)	57 (8.8)	395 (8.4)	0.803
CCB, n (%)	322 (5.7)	61 (9.4)	491 (10.5)	0.450
β-blocker, n (%)	322(5.7)	14 (2.2)	167 (3.6)	0.084
Diuretics, n (%)	322(5.7)	5 (0.8)	42 (0.9)	0.927
Metformin, n (%)	199 (3.5)	34 (5.2)	168 (3.5)	0.043

Abbreviations: Values are mean ± standard deviation or n (%); *RKFD* rapid kidney function decline; *BMI* body mass index; *SBP* systolic blood pressure; *DBP* diastolic blood pressure; *RBC* red blood cell; *WBC* white blood cell; *PLT* platelet; *ALT* alanine aminotransferase; *BUN* urea nitrogen; *Scr* serum creatinine; *eGFR* estimated glomerular filtration rate; *ACEI* angiotensin-converting enzyme inhibitor; *ARB* angiotensin receptor antagonists; *CCB* calcium channel blocker

### Predicting nomogram development

**Fig. 2:**
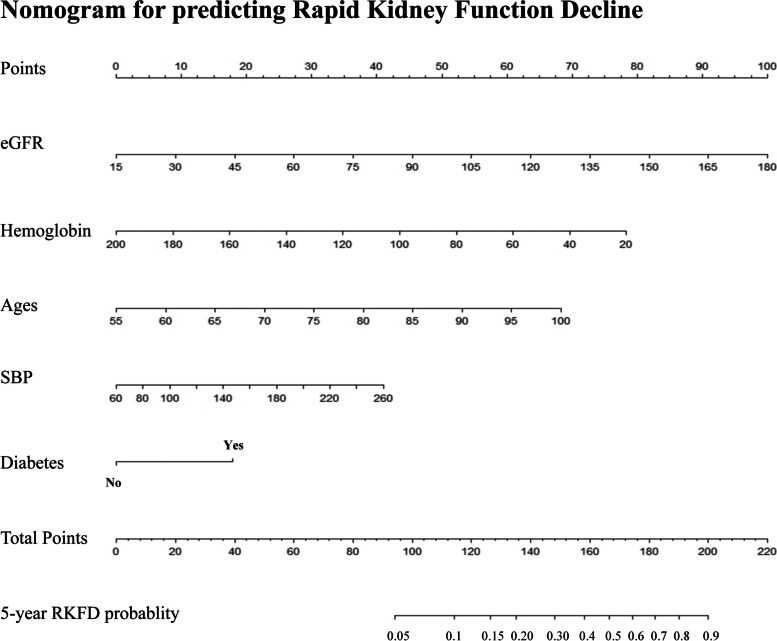
Nomogram to predict the 5-year risk of rapid kidney function decline (RKFD). To use the nomogram, find the position of each variable on the relative axis, draw a line to the points axis for the number of points, add the points derived from all the variables together, and refer to the total points axis to determine the 5-year of RKFD probabilities. eGFR: estimated glomerular filtration rate, SBP: systolic blood pressure

The results of the univariable COX regression analysis are detailed in Table 2. Through multivariable COX regression analysis and a backward stepwise approach, age (HR: 1.056, 95% CI: 1.044–1.068), eGFR (HR: 1.022, 95% CI: 1.019–1.025), hemoglobin (HR: 0.985, 95% CI: 0.98–0.989), SBP (HR:1.007, 95%CI: 1.004–1.011), and diabetes (HR: 1.902, 95% CI: 1.606–2.252) were selected as predictors of RKFD (Table 2). The nomogram to predict 5-year RKFD risk was then constructed based on these five variables (Fig. 2).

**Table 2:** Univariable and Multivariable Cox Regression Analysis of Rapid Kidney Function Decline

Variables	Univariable analysis		Multivariable analysis	
	Adjusted HR (95%CI)	P value	Adjusted HR (95%CI)	P value
Age, years	1.04 (1.03–1.05)	< 0.001	1.056 (1.044–1.068)	< 0.001
Female	0.72(0.61–0.85)	< 0.001		
Height, cm	0.98 (0.97–0.99)	< 0.001		
BMI, kg/m^2^	1.00 (0.99–1.03)	0.600		
SBP, mmHg	1.01 (1.00–1.01)	< 0.001	1.007 (1.004–1.011)	0.002
Diabetes mellitus	1.99 (1.69–2.35)	< 0.001	1.902 (1.606–2.252)	0.002
Hypertension	1.56 (1.28–1.90)	< 0.001		
eGFR, mL/min/1.73m^2^	1.02 (1.02–1.02)	< 0.001	1.022 (1.019–1.025)	< 0.001
Ever smoking	0.65 (0.52–0.83)	< 0.001		
Ever drinking	0.65 (0.49–0.87)	0.002		
Hemoglobin	0.98 (0.97–0.99)	< 0.001	0.985 (0.98–0.989)	0.002
Cholesterol	0.89 (0.83–0.96)	< 0.010		
Uric acid	1.00 (1.00–1.01)	< 0.001		
ALT	1.00 (1.00–1.00)	0.500		
BUN	0.99 (0.97–1.01)	0.400		
Metformin	2.04 (1.44–2.88)	< 0.001		

Abbreviations: *HR* hazard ratio; *BMI* body mass index; *SBP* systolic blood pressure; *eGFR* estimated glomerular filtration rate; *ALT* alanine aminotransferase, *BUN* blood urea nitrogen

### Validation of the predicting nomogram

**Fig. 3:**
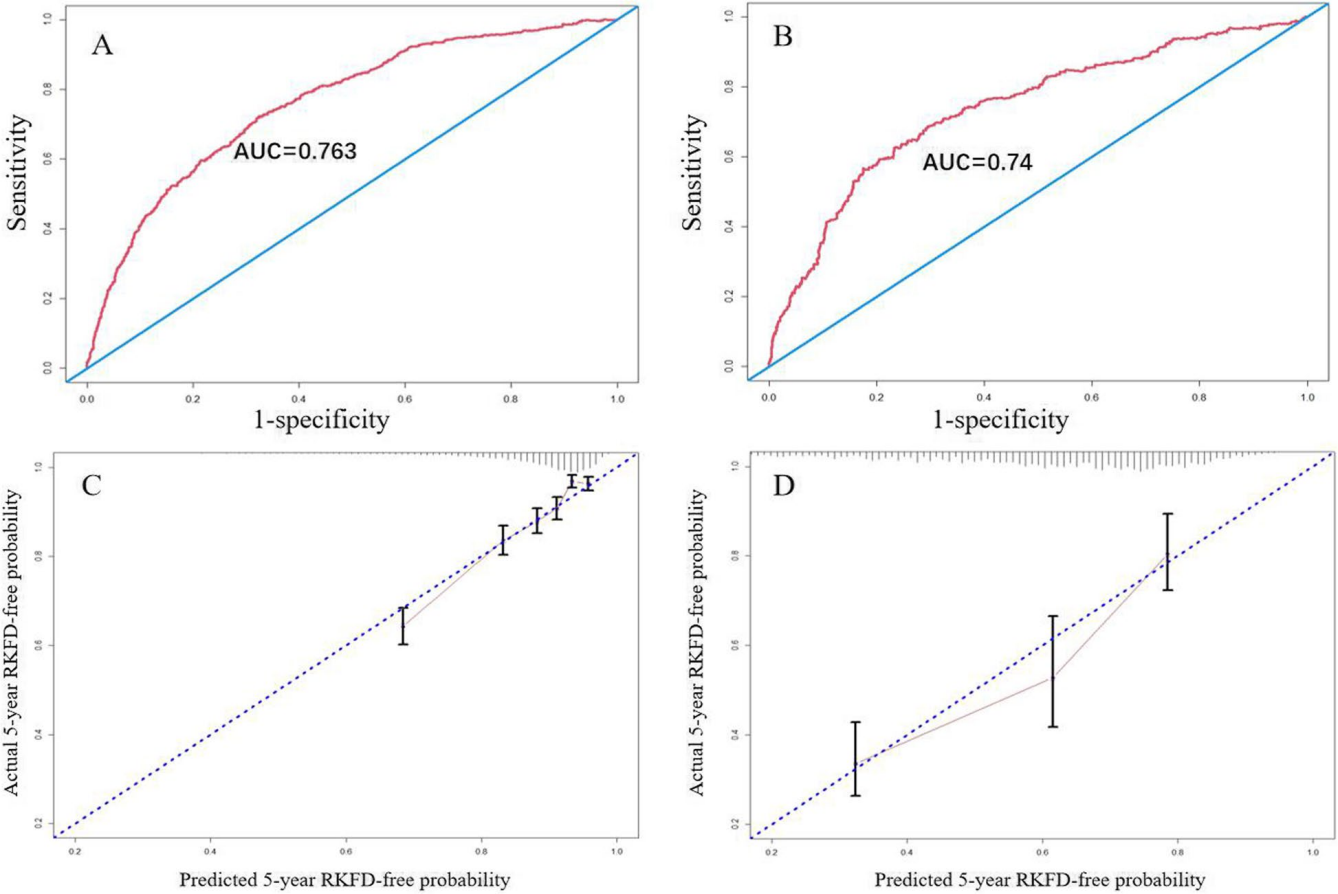
Receiver operating characteristic curves and predictive value validation of the nomogram. The 5-year AUC in the development cohort (**A**) and in the validation cohort (**B**). Validity of the predictive value in the development cohort (**C**) and in the validation cohort (**D**) of the 5-year CKD probability. AUC: area under the curve

In both cohorts, the nomogram demonstrated good discriminative power with C-index of 0.73 in the development cohort and 0.71 in the validation cohort, respectively. The 5-year AUCs were 0.763 in the development cohort and 0.740 in the validation cohort (Fig. 3, A, B).

The calibration plots for the 5-year RKFD indicated that there was good agreement between the actual observations and predictions in both the development cohort and the validation cohort (Fig. 3, C, D). The clinical utility of the nomogram was also confirmed by the decision curve analysis (Supplement figure 1). However, in the subgroup analysis, our prediction model performed well in patients without CKD (AUC: 0.786) but not in those with CKD (AUC: 0.666) (Supplement figure 3).

### Risk stratification of RKFD based on the nomogram scores

**Fig. 4:**
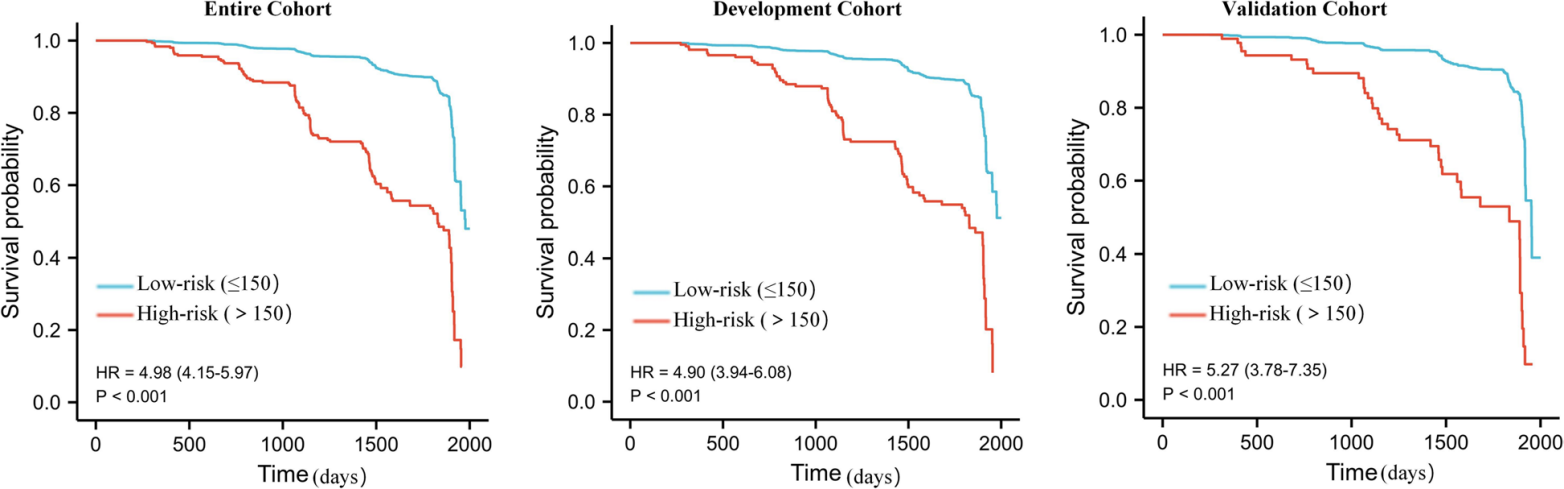
Kaplan-Meier curves of incident rapid kidney function decline for patients in the low- and high-risk groups stratified by total score of 150 points calculated from nomogram. Low-risk group (scores≤150), high-risk group (scores>150)

Based on the predicted 5-year incidence of RKFD in relation to different total nomogram scores, we further divided the participants into 2 score categories: low-risk group (scores≤150, 5-year risk = 11.90%), and high-risk group (scores> 150, 5-year risk = 47.98%). The predicted rates of RKFD in the validation cohort were closed to those in the development cohort within both risk groups (Supplement figure 2). And Kaplan-Meier curves of RKFD for patients in the low- and high-risk groups shown that this risk classification system had a good discriminative power. The incidence of RKFD was significantly higher in the high-risk group (Fig. 4).

## Discussion

The present study maybe the first to develop a nomogram for the prediction of 5-year RKFD among the population at risk of cardiovascular disease. Our simple nomogram including 5 predictors: age, eGFR, hemoglobin, SBP, and diabetes, demonstrated good discrimination and calibration. With the cut-off point of 150, we may easily identify individuals at high risk of RKFD.

Rapid declining kidney function is associated with higher risk of myocardial infarction (MI), heart failure (HF), stroke, and peripheral arterial disease (PAD) among patients with or without CKD [10], especially for the elderly who are even more likely to suffer from CVD and CKD. The elderly and adults with chronic disease (hypertension and diabetes) are currently the key monitoring population for the China National Basic Public Health Service. Therefore, to quickly identify individuals with rapidly declining renal function among this population is very meaningful, which may help reducing the incidence of CVD and improve their quality of life. As for the definition of “RKFD”, some studies defined RKFD as annual eGFR declines of 3 ml/min per 1.73m^2^ [10, 11] or 5 ml/min per 1.73m^2^ [12]. Recently, a Japanese study among healthy subjects reported that eGFR decline non-linearly altered according to age [13]. Compared with these absolute values change, the percentage decrease of eGFR seems to be more personalized in defining the RKFD. Moreover, the ARIC cohort study including 13,029 participants showed that patients with annual decline (annual decline ≥5.65%) in eGFR was at significantly greater risk for coronary heart disease [3]. In year 2014, the NKF and FDA published a series article to suggest that eGFR declines of 40% could be an effective surrogate endpoint in the clinical trials [14]. Therefore, wo chose eGFR declines of 40% to be the definition of RKFD in this study.

In our study, we identified 5 independent predictors of RKFD: age, baseline eGFR, hemoglobin concentration, SBP, and diabetes. This finding was partly consistent with those of prior studies [4, 8, 15–18]. In a community-based retrospective cohort study among 51,938 adults who underwent annual medical examinations between 1999 and 2013, aging, higher SBP, proteinuria, and smoking were related to faster loss of kidney function [16]. A prospective observational cohort study - KNOW-CKD Study [17] and a study based on Chinese population [12] also found that SBP had a greater association with adverse kidney outcomes than DBP. These findings clearly emphasize that SBP is an important correlated factor for faster loss of kidney function. Anemia is also an independent risk factor for worsening kidney function among the middle-aged and elderly population [18]. As we all known, CKD often complicated with anemia mainly due to iron deficiency and erythropoietin deficiency [19, 20]. However, on the other side, anemia could also contribute to kidney function decline, which may be due to hypoxia and/or increased oxidative stress [21]. Anemia decreases the oxygen delivery to tissues and thus affects organ function including kidney and heart [18]. The stage of baseline eGFR has been proven to be associated with incident CKD [8, 22], and we further found that the baseline eGFR could also contribute to the rapid eGFR decline (HR = 1.022, 95%CI 1.019–1.025), and this finding further confirmed that the baseline value of eGFR is essential for future kidney function development [23]. There is no doubt that diabetes is an independent risk factor for kidney function decline [24].

We noticed that, in the univariate analysis, there were some phenomena that were different from clinical cognition, such as lower serum creatinine, cholesterol, and uric acid, higher eGFR, lower smoking and drinking rate in patients developing RKFD. For the lower creatinine and higher eGFR in the patients developing RKFD, it may be caused by the endpoint of our study, which was defined by the percentage declined in eGFR. For individuals with better baseline renal function, the space for the decline in renal function was greater than that of people with baseline renal insufficiency. As for uric acid, in the community physical examination in 2015, uric acid was not routinely tested, so the rate of missing data was over 22%. Therefore, the uric acid was not included in the multivariable Cox regression analysis. And for the smoking and drinking, one reason may be that individuals who often smoking or drinking were relatively young, and, we could find that smoking and drinking were excluded after adjusting for other influencing factors through multivariate COX regression analysis. In the further research, we would carry out prospective research to improve the quality of our baseline data and verify the model.

### Strengths and limitations

To our knowledge, this might be the first study to develop a nomogram for the prediction of 5-year risk of RKFD in a high CVD risk population. Our nomogram derived from clinical factors that are readily accessible in primary care. And it enables us to identify individuals at risk of RKFD readily and to take action in time. Compared to the previous studies with endpoints like CKD or end stage renal disease, eGFR declining more than 40% demonstrates the dynamic change of the kidney function among individuals with or without CKD, it is more meaningful for the primary care.

This study has several limitations. First, most participants were lack of albuminuria data at baseline. Positive proteinuria is related to increasing risk of kidney disease and death [25], whereas a large proportion of people with kidney disease dot not have albuminuria [26]. And in the Chinese primary care setting, proteinuria is usually measured in patients with DM, so our nomogram maybe more available for the community health institution. Second, though we have recorded past history in every annual physical examinations, we have difficulty in recognizing minority emergencies episodes, such as acute glomerulonephritis and acute kidney injury caused by other nephrotoxic drugs between physical examinations. Third, since the nomogram developed in our study was only based on routinely collected data, its performance among patients with CKD was not so good as its performance among those without CKD. Its predictive accuracy in CKD patients may be further enhanced by adding newly identified biomarkers or geno-type data of RKFD in the future [27].

## Conclusions

Constructed based on the demographic, clinical and laboratory variables from electronic health records, our nomogram is clinically applicable and easy to use in primary care setting. Although our nomogram performed well in our validation cohort, further external validations are needed before clinical generalization.

## Supplementary Information

**Additional file 1.**

## Data Availability

The datasets generated during and analyzed during the current study are not publicly available due to there was a confidentiality agreement signed with the partner hospitals, but the datasets are available from the corresponding author on reasonable request.
