# Supplementary material for: Nomogram to predict rapid kidney function decline in population at risk of cardiovascular disease
Source: BMC Nephrol. 2022 Feb 10;23:62. doi: 10.1186/s12882-022-02696-9 (PMC8830119; doi:10.1186/s12882-022-02696-9)
Supplement: Supplementary file 1 — Additional file 1. [file 12882_2022_2696_MOESM1_ESM.docx]

**Supplemental material**

**Table S1**. Baseline characteristics of the entire cohort.

| Variables | Overall  (n = 8455) | Missing data (%) | Development cohort (n = 5645) | Validation  cohort (n = 2810) | P value |
| --- | --- | --- | --- | --- | --- |
| Age, years | 67.69±6.74 | 0 (0) | 67.66±6.70 | 67.77±6.82 | 0.481 |
| Female, n (%) | 5211 (61.6) | 0 (0) | 3468 (61.4) | 1743 (62.0) | 0.614 |
| Height, cm | 155.96±8.05 | 0 (0.05) | 155.89±8.02 | 156.10±8.11 | 0.255 |
| Weight, kg | 59.56±10.38 | 4 (0.05) | 59.51±10.33 | 59.66±10.50 | 0.536 |
| BMI, kg/m^2^ | 24.44±3.60 | 4 (0.05) | 24.45±3.59 | 24.44±3.63 | 0.913 |
| Waist, cm | 85.02±9.39 | 35 (0.4) | 84.94±9.41 | 85.16±9.36 | 0.319 |
| SBP, mmHg | 146.34±20.39 | 15 (0.1) | 146.20±20.40 | 146.63±20.38 | 0.353 |
| DBP, mmHg | 81.83±11.35 | 21 (0.2) | 81.72±11.32 | 82.06±11.42 | 0.19 |
| Diabetes mellitus, n (%) | 1681 (20.4) | 0 (0) | 1090 (19.9) | 591 (21.5) | 0.085 |
| Hypertension, n (%) | 6355 (75.2) | 0 (0) | 4232 (75.0) | 2123 (75.6) | 0.577 |
| Ever smoking, n (%) | 1504 (17.8) | 2 (0.2) | 1005 (17.8) | 499 (17.8) | 0.986 |
| Ever drinking, n (%) | 1026 (12.1) | 0 (0) | 678 (12.0) | 348 (12.4) | 0.645 |
| Exercise |  | 6 (0.07) |  |  |  |
| Never, n (%) | 3905 (46.2) |  | 2595 (46.0) | 1310 (46.7) | 0.581 |
| Once a week, n (%) | 1326 (15.7) |  | 879 (15.6) | 447 (15.9) |  |
| Few times a week, n (%) | 444 (5.3) |  | 289 (5.1) | 155 (5.5) |  |
| Daily, n (%) | 2774 (32.8) |  | 1878 (33.3) | 896 (31.9) |  |
| **Laboratory examination** |  |  |  |  |  |
| Fasting glucose, mmol/L | 5.29±1.78 | 8 (0.08) | 4.87 [4.41, 5.51] | 4.87 [4.40, 5.58] | 0.471 |
| RBC, 10^12^/L | 4.71±0.59 | 142 (1.68) | 4.64 [4.32, 5.02] | 4.63 [4.31, 5.00] | 0.478 |
| Hemoglobin, g/L | 136.03±15.58 | 90(1.06) | 136.00 [127.00, 146.00] | 136.00 [127.00, 145.00] | 0.682 |
| WBC, 10^9^/L | 6.77±1.85 | 39 (0.46) | 6.60 [5.60, 7.70] | 6.60 [5.60, 7.70] | 0.739 |
| PLT, 10^9^/L | 215.56±57.93 | 24 (0.29) | 211.00 [177.00, 249.00] | 211.00 [177.00, 250.00] | 0.556 |
| ALT, U/L | 26.87±20.22 | 18(0.21) | 22.70 [17.30, 30.50] | 21.90 [16.83, 30.10] | 0.064 |
| BUN, mmol/L | 5.96±9.90 | 36 (0.43) | 5.40 [4.60, 6.40] | 5.40 [4.53, 6.50] | 0.942 |
| Cholesterol, mmol/L | 5.40±1.18 | 15 (0.18) | 5.33 [4.61, 6.08] | 5.33 [4.61, 6.09] | 0.93 |
| Triglyceride, mmol/L | 1.72±1.12 | 15 (0.18) | 1.42 [0.99, 2.08] | 1.41 [0.98, 2.11] | 0.681 |
| Uric acid, umol/L | 377.57±110.76 | 1749(20.69) | 367.20 [298.30, 446.95] | 367.00 [301.20, 443.10] | 0.971 |
| Scr, umol/L | 70.79±24.60 | 0 (0) | 66.10 [54.91, 80.60] | 66.22 [55.20, 81.60] | 0.387 |
| eGFR, mL/min/1.73m^2^ | 93.92±26.64 | 0 (0) | 92.35 [76.87, 109.47] | 91.60 [76.06, 109.55] | 0.204 |
| **Medications** |  |  |  |  |  |
| ACEI/ARB, n (%) | 668 (8.4) | 464 (5.49) | 452 (8.5) | 216 (8.1) | 0.581 |
| CCB, n (%) | 813 (10.2) | 465(5.5) | 552 (10.4) | 261 (9.8) | 0.438 |
| β-blocker, n (%) | 269 (3.4) | 465(5.5) | 181 (3.4) | 88 (3.3) | 0.865 |
| Diuretics, n (%) | 66 (0.8) | 465(5.5) | 47 (0.9) | 19 (0.7) | 0.507 |
| Metformin, n (%) | 302 (3.7) | 295 (3.5) | 202 (3.7) | 100 (3.7) | 1 |

Abbreviations: BMI, body mass index; SBP, systolic blood pressure; DBP, diastolic blood pressure; RBC, red blood cell; WBC, white blood cell; PLT, platelet; ALT, alanine aminotransferase; BUN, urea nitrogen ; Scr, serum creatinine; eGFR, estimated glomerular filtration rate; ACEI, angiotensin-converting enzyme inhibitor; ARB, angiotensin receptor antagonists; CCB, calcium channel blocker.


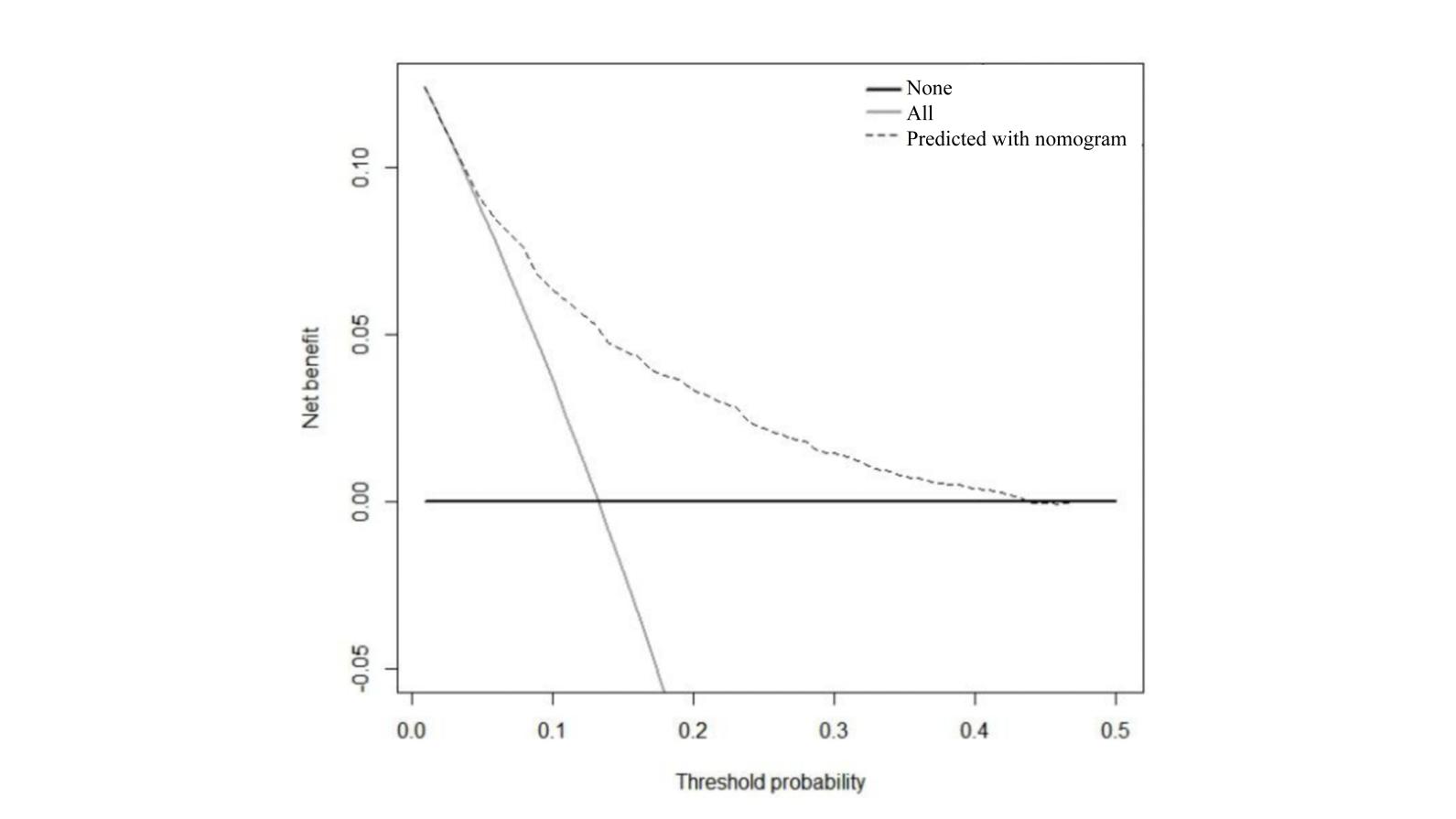


**Supplement figure 1**: Decision curve analysis (DCA) for the rapid kidney function decline (RKFD) risk prediction model. The dotted line is located at the upper right of the horizontal and vertical threshold line, which represents the clinical benefit of using nomogram to predict RKFD.


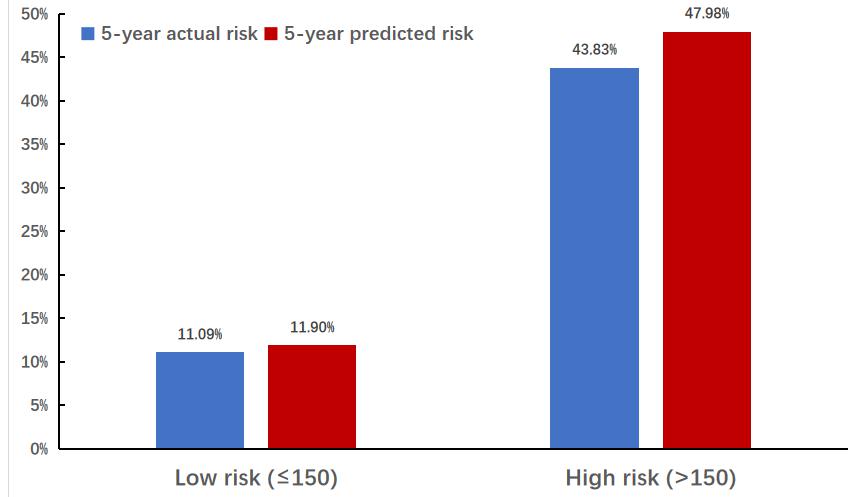


**Supplement figure 2**: Risk stratification of 5-year rapid kidney function decline (RKFD) based on the nomogram scores. Low-risk group (scores≤150), high-risk group (scores>150). The predicted rates of RKFD in the validation cohort were closed to those in the development cohort inside both of the low and high-risk groups.


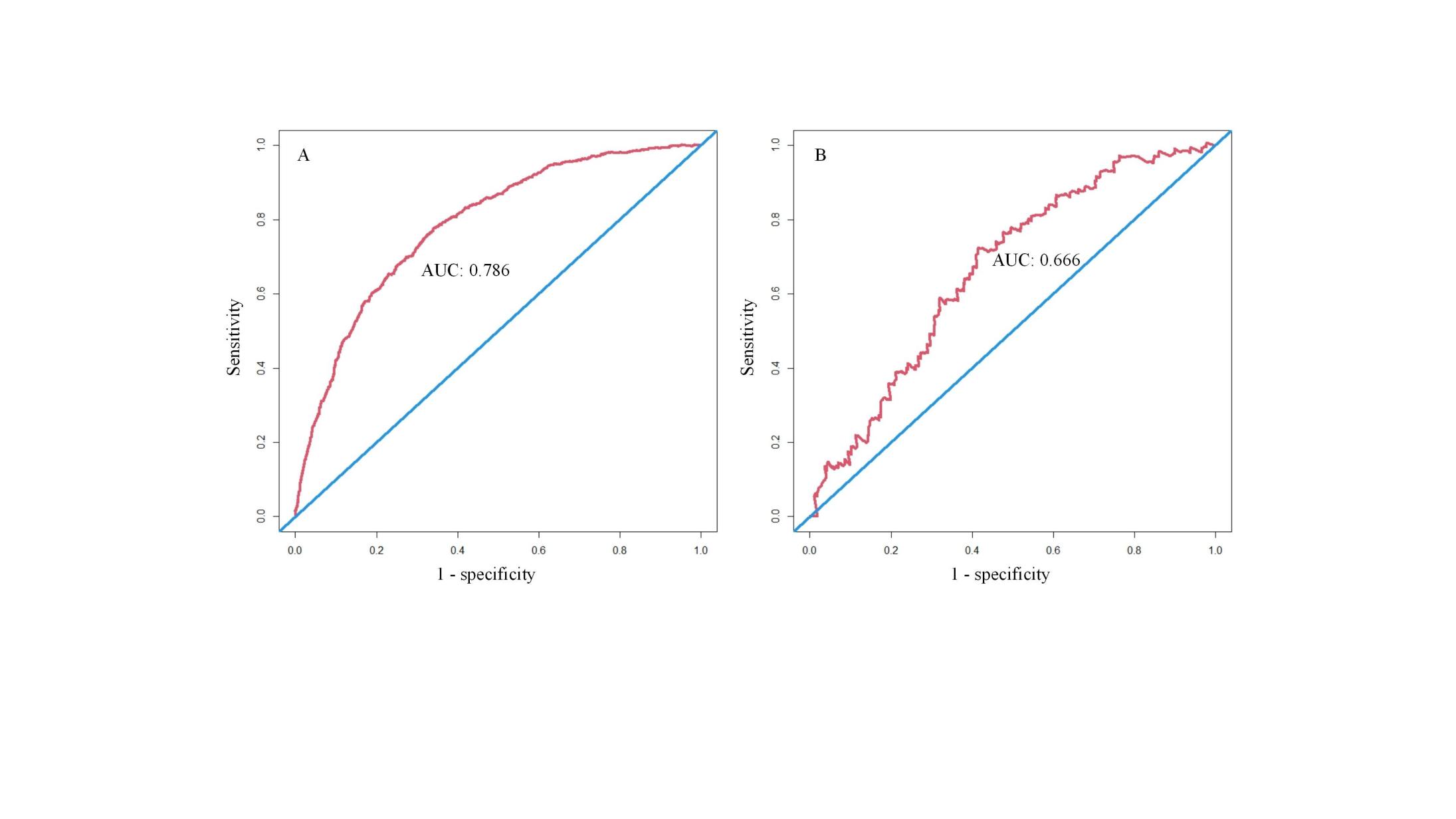


**Supplement figure 3**: Receiver operating characteristic curves and predictive value validation of the nomogram for patients without CKD(A) and with CKD (B). AUC: area under the curve.
